# Comparative Study on A Novel Pathogen of European Seabass. Diversity of *Aeromonas veronii* in the Aegean Sea

**DOI:** 10.3390/microorganisms7110504

**Published:** 2019-10-29

**Authors:** Maria Smyrli, Adriana Triga, Nancy Dourala, Panos Varvarigos, Michael Pavlidis, Viet Ha Quoc, Pantelis Katharios

**Affiliations:** 1 Institute of Marine Biology, Biotechnology and Aquaculture, Hellenic Centre for Marine Research, Heraklion, 71500 Crete, Greece; msmyrli@hcmr.gr (M.S.); triga@hcmr.gr (A.T.); qvha@hcmr.gr (V.H.Q.); 2 Department of Biology, University of Crete, Heraklion, 70013 Crete, Greece; pavlidis@biology.uoc.gr; 3 Fish Pathology Department, Selonda Aquaculture, 15125 Athens, Greece; adourala@otenet.gr; 4 VETCARETM, 11528 Athens, Greece; info@vetcare.gr

**Keywords:** *Aeromonas veronii*, European seabass, Aegean Sea, Mediterranean, comparative genomics, virulence, OMPs

## Abstract

*Aeromonas veronii* is an emerging pathogen causing severe pathology and mortalities in European seabass aquaculture in the Aegean Sea, Mediterranean. More than 50 strains of the pathogen were characterized biochemically and genetically in order to study the epidemiology of the disease, as well as the phylogeny and virulence of the bacterium. Based on the phenotypic characteristics, the isolates form three groups consisting of: (a) the West Aegean Sea, non-motile, non-pigment-producing strains, (b) the West Aegean Sea, motile, and pigment-producing strains and (c) the East Aegean Sea motile strains that produce minute amounts of pigment. All strains were highly similar at the genomic level; however, the pattern of West/East geographic origin was reflected in biochemical properties, in general genomic level comparison and in the putative virulent factors studied. Type VI secretion system was not detected in the western strains. The outer membrane protein (OMP) profile which contains proteins that are putative antigenic factors, was very similar between strains from the different areas. Although most of the OMPs were detected in all strains with great sequence similarity, diversification according to geographic origin was evident in known antigenic factors such as the maltoporin LamB. A systematic comparative analysis of the strains is presented and discussed in view of the emergence of *A. veronii* as a significant pathogen for the Mediterranean aquaculture.

## 1. Introduction

Mesophilic, motile *Aeromonas* spp. are responsible for ulcerative, hemorrhagic, and septicemic infections in freshwater and ornamental fish [1]. While *A. hydrophila* is the most cited, modern diagnostic tools [2] have resulted in the identification of many more species implicated in disease development including A. *veronii*, *A. bestiarum*, *A. caviae*, *A. jandaei*, *A. piscicola*, *A. schubertii*, and *A. sobria*. Amongst them, *A. veronii* is increasingly gaining importance as a serious pathogen for the aquaculture industry. Outbreaks accompanied by significant losses have been reported in African catfish (*Clarias gariepinus*), rajputi (*Puntius gonionotus*), rui (*Labeo rohita*), catla (*Catla catla*), and shole (*Channas triatus*) farmed in Bangladesh [3], in Chinese longsnout catfish (*Leiocassis longirostris*) [4] loach (*Misgurnus anguillicaudatus*), [5] and cyprinid fish [6] farmed in China and in ayu (*Plecoglossus altivelis*) farmed in Japan [7]. In addition to aquaculture, *A. veronii* has also been reported to cause disease in ornamental fishes [8].

During the past decade, A. *veronii* bv. *sobria* has become extremely problematic in the culture of European seabass (*Dicentrarchus labrax*) in Greece. The disease first appeared in 2008 affecting a single fish farm in Central Greece [9]. While it is still present in the specific farm, more farms in distant areas are also affected. At the beginning, the disease affected mainly fish reaching the commercial size (>200 g), but lately it has been reported to affect also younger fish with weight lower than 50 g. Outbreaks occur during the warm months of the year, when water temperature is over 21 °C. Cumulative mortality during outbreaks reaches >50%, if it is not treated with antibiotics and it is a major concern for the producers in the affected areas. 

Affected fish appear lethargic with no appetite and in progressed stages of the disease they have an icteric appearance due to the highly haemolytic nature of the pathogen. Internally, multiple abscesses are usually found in the spleen and the kidney. Interestingly, the pathogen affects exclusively the European seabass and even during severe outbreaks, other fish species like the gilthead seabream (*Sparus aurata*), red porgy (*Pagrus pagrus*), and sharpsnout seabream (*Diplodus puntazzo*), which are cultured in adjacent sea cages of the affected areas are not infected (personal observations).

Over the past decade, we have collected a large number of *A. veronii* strains from European seabass of Greek aquaculture farms during disease outbreaks at various time points during the year. Here, we present a comparative study of 50 *A. veronii* clinical strains from different geographic areas. Biochemical diversity and phylogenetic relationships were assessed. Comparative genomic analysis was conducted to assess similarity at the genome level and to study putative virulence factors that may have contributed to pathogenicity. Pathogenicity was also tested in vivo using zebrafish as a model. We followed the principles of reverse vaccinology using the draft genomes of nine *A. veronii* strains, representatives of the geographic location, the phenotype, and the year of isolation in order to assess the antigenic diversity of the species in Greece. Through this study we identified and compared outer membrane proteins (OMPs) to establish the basis for effective strain selection for a future vaccine for seabass in the Aegean Sea. The final goal of this work was to acquire knowledge on the diversity of a novel pathogen and contribute to the management of a very important emerging disease for the Mediterranean aquaculture industry.

## 2. Materials and Methods

Localities included in the study were selected based on reports from the fish vets of the affected farms for aeromonad-suspected morbidity and mortality. Extended sampling was conducted in the area of Argolikos Bay where the disease was first described [9]. Microbial screenings on diseased fish farmed in sea-cages, were conducted at various times (2009–2019) including periods of disease outbreaks but also periods when fish showed no signs of the disease. Only the positive for *Aeromonas* spp. samplings are presented here. In the study we have also included samples from disease outbreaks in the ornamental freshwater fish Green swordtail (*Xiphophorus helleri*) farmed in North/East Greece and in zebrafish (*Danio rerio*) from the experimental facilities of the Biology Department of the University of Crete, Greece. Detailed information on the samplings conducted is presented in Table 1.

Samples of fish and bacteria were either collected on site or were sent and processed at the Hellenic Centre for Marine Research (HCMR). Fish underwent full necropsy and clinical signs of disease were recorded when present. Bacteria were isolated from the kidney of fish on Trypticase soy agar (TSA) (Trafalgar Scientific, Leicester, UK) supplemented with salt (2% NaCl) and on the selective for *Aeromonas* spp. Aeromonas isolation agar (AIA) (Sigma-Aldrich, St. Louis, MO, USA) supplemented with ampicillin. Aeromonads detection was based on growth on AIA and positive PCR reaction for the 16S-23S rRNA intergenic spacer region using genus-specific primers for *Aeromonas* spp. [10]. Subsequently, identification to genus level was achieved by PCR amplification of the extracellular lipase Glycerophospholipid-cholesterol acyltransferase (GCAT) gene [11]. Only positive isolates (1–7 per sampling) were further analysed by random selection. For identification to species level, the gene of B-subunit of DNA gyrase (*gyr*B) was amplified according to reference [12] and sequenced in ABI3730xl sequencer based on the protocol of BigDye Terminators 3.1 (Applied Biosystems, Thermo Fisher Scientific, Carlsbad, CA, USA). Sequences were deposited to GenBank under the Accession Numbers: MN193961-MN193984 and MN193987-MN194010. The obtained sequences were compared in GenBank using the NCBI BLAST algorithms. Phylogenetic analysis was conducted using the sequences of *gyr*B gene produced herein and sequences from all the *Aeromonas* spp. known up to date. Alignment was performed in Clustal W [13]. Genetic distances were estimated and phylogenetic relationships were examined with neighbor-joining (NJ) analyses [14] in MEGA X [15] under Tamura-Nei [16] model of evolution. The confidence of tree nodes was tested by the bootstrap analysis with 1000 replicates.

Colony morphology was observed on TSA. Motility was tested on motility, indole, and ornithine (MIO) medium (Sigma-Aldrich). Pigment production was tested on TSA and Mueller-Hinton agar (MH) (Trafalgar Scientific, Leicester, England) after incubation for 24–72 h, as well as their corresponding broths after incubation for 7 d.

Biochemical characterization was conducted with the commercial kits BIOLOG GEN III Microplate (BIOLOG) (Biolog, Hayward, CA, USA) and API 20E (bioMérieux Hellas S.A, Athens, Greece). Results were recorded after 24 h incubation for API 20E and after 48 h incubation for BIOLOG. BIOLOG and API 20E were also used for identification to genus level [2]. Catalase reaction was tested separately. The TSA and MH mediums were supplemented with 0.5% NaCl. All tests were performed at 25 °C. The type strains LMG 3785 (*A. veronii* bv. *sobria*) and LMG 9075 (A. *veronii* bv. *veronii*) were included in all tests as reference strains. Hemolytic activity was tested on 5% seabass blood agar [17]. Fish blood was taken aseptically from healthy European seabass broodfish maintained in the aquaculture facilities of HCMR. Results were recorded at 24 and 48 h incubation at 25 °C. Susceptibility to antibacterial agents was assessed by the disk diffusion method [18] using 6-mm commercial disks (Oxoid, Thermo Fisher Scientific) on MH agar supplemented with 0.5% NaCl. The inhibition diameter was recorded after incubation for 48 h at 22–25 °C. Inhibition diameters were relatively set by Enterobacteriaceae and compared with relevant literature [19,20].

Morphology of the bacteria with emphasis on the cellular components related to motility (flagellum, fimbria, pili) was studied with transmission and scanning electron microscopy. Bacteria were grown for 6 hours in TSB and preserved in 2.5% glutaraldehyde in phosphate buffer. Bacteria preparations were negatively stained with 4% (w/v) uranyl acetate (pH 7.2) and observed with a JOEL JEM2100 transmission electron microscope (TEM) operated at 80 kV. Samples for scanning electron microscope (SEM) were washed with sodium cacodylate buffer, post fixed with OsO_4_, and dehydrated in an ascending alcohol series, mounted on stubs, and sputter coated with gold-palladium. Bacteria were viewed using a JEOL JSM-6390LV scanning electronic microscope at 20 kV. Both TEM and SEM were conducted at the Electron Microscopy Laboratory of the University of Crete.

Hierarchical cluster analysis was conducted on the phenotypic and biochemical data tested including the API 20E, BIOLOG’s and catalase reactions, motility, pigment production, and β-hemolysis (119 characters). Analysis was conducted in IBM SPSS Statistics software using between-groups linkage method with Chi-Square measure.

Nine strains from seabass and one strain from *X. helleri* were subjected to whole genome sequencing. Paired-end sequencing was performed using an Illumina MiSeq platform (Illumina, Inc. San Diego, CA, USA). For the de novo assembly, MaSuRCa genome assembler and SPAdes were used [21]. Quality of the assembled genome sequences was assessed by Benchmarking Universal Single-Copy Orthologs (BUSCO) [22,23]. Gene identification and annotation were done using the NCBI Prokaryotic Genome Annotation Pipeline [24] and RAST [25]. The bacterial genomes were also analyzed in the platform of the Pathosystems Resource Integration Center (PATRIC) [26]. Genomes were deposited to GenBank under the Accession Numbers: NZ_NMUR00000000.1 (NS), NZ_NMUS00000000.1 (PDB), NZ_NPKE00000000.1 (NS 2), NZ_NPKC00000000.1 (NS 6.15.2), NZ_NQMB00000000.1 (NS 13), NZ_NQMC00000000.1 (NS 22), NZ_NNSE00000000.1 (AG 5.28.6), NZ_NNSF00000000.1 (VCK 1), NZ_NPKD00000000.1 (BIOO50A), NZ_SSUX00000000.1 (XU 1).

Multilocus sequence typing (MLST) analysis was conducted based on the Web-based MLST sequence database (https://pubmlst.org/aeromonas/) [27,28]. The allele sequences for the six genetic loci (*gyr*B, *gro*L, *glt*A, *met*G, *pps*A, and *rec*A), were retrieved from the ten *Aeromonas* genomes. The ST profile of each strain was created and compared in the database with other 645 different STs (July 2019). The complete sequences of the six genes were used a) to estimate the genetic distances among seabass strains and with the strains XU 1 and B565 (*A. veronii*) and b) for phylogenetic analyses (NJ) following concatenation. Analyses were performed in MEGA X [15] under the Tamura-Nei [16] model of evolution. The confidence of tree nodes was tested by the bootstrap analysis with 1000 replicates.

The similarity of the nine genomes isolated from seabass with each other and with other *Aeromonas* spp. was assessed using Average Nucleotide Identity by Orthology [29] with Ortho-ANI software. Comparisons with other *A. veronii* genomes from different hosts and isolation sources, and the strain XU 1 from *X. helleri* described here, and the strain A449 of *A. salmonicida* subsp. *salmonicida* and the type strain ATCC 7966 of *A. hydrophila* subsp. *hydrophila*, were also made.

Single nucleotide polymorphisms (SNPs) analysis was done according to [30]. Briefly, SNPs were determined using CSI phylogeny [31,32] available on the CGE (www.genomicepidemiology.org). The paired-end reads of the nine *Aeromonas* strains were mapped to the reference chromosome of the strain B565. Qualified SNPs were determined when fulfilling the following criteria: (1) A minimum distance of 10 bps between each SNP, (2) a minimum of 10% of the relative depth at SNP positions, (3) the mapping quality was above 25, (4) the SNP quality was more than 30, and (5) all indels were excluded. The SNPs from each genome were concatenated to a single alignment corresponding to the position of the reference genome. The concatenated sequences were subjected to maximum likelihood analysis using MEGA X.

The profile of the outer membrane proteins (OMPs) was first studied in the randomly selected strain VCK 1. The proteome (as predicted by the genomic analysis) of VCK 1 was analyzed in PSORTb v.3.0, a tool for subcellular localization of proteins [33]. The detected OMPs, were further analyzed with Signal IP v.5 in order to detect signal peptides (and cleavage sites) of proteins targeting the outer membrane [34]. TOPCONS2 was used to predict the topology of alpha-helical transmembrane proteins [35] and PRED-TMBB2 to predict the topology of beta-barrel proteins [36]. The set of proteins identified in this way for VCK 1 were also located in the other *A. veronii* strains from seabass, and from strains XU 1 from *X. helleri* and strain B565 through mapping in Geneious v.9.1.6. Similarity of these proteins was calculated in Geneious using protein distance following alignment of the aminoacidic sequences with the MUSCLE algorithm.

Genomic Islands (GIs) were predicted for the nine isolates from seabass through the online platform Island Viewer v.3 [37] that includes three methods, IslandPick, IslandPath-DIMOB, and SIGI-HMM, for prediction and detection of GIs. Prophage sequences were identified by the PHASTER web server [38]. Virulence genes were detected manually and using the PATRIC annotation platform [26], which combines three databases; PATRIC_VF, VFDB, and VICTORS. Incomplete coding sequences (CDS) located at the edge of contigs were excluded. Special focus was given to the secretion systems, flagellar proteins and toxins, which were studied using alignment with the respective gene clusters identified in the *Aeromonas* strains of the current study. Alignment was conducted with CLUSTALW and Mauve [39] in Geneious v.9.1.6. Antibiotic Resistance genes were predicted by the Resistance Gene Identifier (RGI) Software of the CARD platform [40].

Adult zebrafish (*Danio rerio*) (mean weight: 0.3 g) were used for assessing the virulence of the nine *A. veronii* strains from seabass. Fish were acclimatized for at least ten days before handling. Water and room temperature were kept at 25 °C. For each strain, 50 fish were distributed in five 5-L tanks each containing 10 individuals. Five doses (10^8^–10^4^ cfu/fish) of live bacteria diluted in sterile PBS were injected in the experimental groups while the control group was injected with sterile PBS. The injection (10 μL/fish) was conducted using a Hamilton micro-syringe following anesthesia (MS222). The fish were monitored over a five-day period, dead fish were removed daily, and mortalities were recorded. LD50 value was estimated by the dose-response curve at 24 and 48 h post injection using Probit analysis in SPSS Statistics. The procedure was performed at the University of Crete which has licensed designated facilities for experimentation with animals (registration number: EL-BIOexp-10) and the protocol was approved by the General Directorate of Regional Agricultural Economy and Veterinary Services of the Region of Crete (License number: 147115/17-07-2017).

## 3. Results

### 3.1. Aeromonad Detection and Prevalence

Aeromonads where detected in fish exhibiting the main clinical signs (loss of appetite, icteric appearance, petechial hemorrhages, etc.) as described previously [9] but also in fish exhibiting only darkening, loss of appetite, and reduced growth (e.g. sampling 4) as well as in younger fish below 50 g. Aeromonads were detected in diseased fish in temperature over 18 °C (March to December) while outbreaks occurred in summer in water temperature mainly over 23 °C. Over one hundred isolates were identified as *Aeromonas* spp. with the methodology described before. From the *Aeromonas* spp. isolated from seabass, 1–7 per sampling (53 in total from seabass) were randomly selected for further characterization and identification to species level together with two isolates from the freshwater fish species (Table 2). Prevalence of aeromonads on diseased fish ranged between 11%–75% (Table 2). Note that in the cases that the bacteria were collected on site by the fish vets, each isolate corresponds to one diseased fish. In these cases, the total number of fish examined is not available and therefore prevalence cannot be estimated.

### 3.2. Molecular Identification and Phylogeny

For identification to species level, the *gyr*B gene was amplified and sequenced for all 55 isolates studied. In total four *Aeromonas* species were identified through BLAST correlation (*p* = 99%), named *A. bivalvium*, *A. media*, *A. salmonicida* and *A. veronii*. Among them, *A. veronii* was the most common aeromonad species (50/53 identified isolates) from diseased seabass. *Aeromonas media* and *A. salmonicida,* were isolated together from one fish (sampling 15) and *A. bivalvium* was isolated together with *A. veronii* from another fish (sampling 17). The isolates XU 1 and Z 1 were also identified as *A. veronii* through BLAST.

Sequences (1004 bp) from the 53 seabass isolates were included in the phylogenetic analyses. Fifty isolates from seabass and the isolates XU 1 and Z 1 were grouped together and formed a monophyletic clade with *A. veronii* in NJ analyses based on *gyr*B sequences (Figure S1). The mean genetic distance between the *A. veronii* isolates from seabass was 0%. The mean genetic distance in the *A. veronii* clade was calculated to 0.015%.

### 3.3. Phenotypic and Biochemical Characterization and Identification

All *Aeromonas* spp. isolates appeared green on AIA (did not produce acid from xylose) and formed full grown colonies after 48 h incubation on AIA/TSA. All but two (NS and NS 13) isolates from seabass were motile. The *A. veronii* isolates form West Aegean Sea (except for NS and NS 13) produced dark brown pigment on TSA and MH agar after 36–48 h incubation. The *A. veronii* isolates from East Aegean produced a light brown pigment mainly visible on MH agar. Those were recorded as positive-intermediate (I) for this attribute in the current study. No pigment production was observed in broth media. The *A. veronii* isolates XU 1 and Z 1 from green swordtail and zebrafish respectively, were both motile and non-pigment-producing as well as the type strains LMG 3785 (*A. veronii* bv. *sobria*) and LMG 9075 (*A. veronii* bv. *veronii*). Results on motility and pigment production are presented in Table 3.

All *A. veronii* isolates from seabass were catalase and oxidase positive, able to ferment D-glucose. All were negative for indole and H_2_S production, did not hydrolyze urea, and did not ferment amygdalin, arabinose, melibiose, inositol, sorbitol, or rhamnose. All fermented mannitol and sucrose and all but one (BIOO50A) were negative for Ornithine decarboxylase. The majority of western strains were negative for β-galactosidase while most of the eastern ones were positive. Reactions for ADH and LDC were almost exclusively positive for western strains while for the eastern ones variability was evident. Beta-hemolysis was clearly demonstrated on seabass blood agar for all isolates tested, after incubation for 24 h at 25 °C. The results reactions of API 20E, catalase, and beta-hemolysis test are presented in Table 3.

Identification in BIOLOG’s database (Microlog 5.2) identified the seabass isolates as *A. hydrophila*-like, DNA group 2, or in few occasions, as *A. sobria* (e.g., PDB). All grew in pH 6 and 1% NaCl but growth in pH 5 was variable and only few were able to grow in salinity ≥4% NaCl. All the western isolates and none but two (T04-D and AG-5.34.6) eastern ones fermented trehalose. Additionally, none but three (AG-5.34.6, NS 31.2.1, and NS 33.1) were able to produce acid from D-arabitol. Western/eastern isolates differed also in the metabolism of β-Methyl-D-Glucoside (−/+) and D-Gluconic acid (+/−). Results of BIOLOG’s reactions are presented in Table S1. Intermediate reactions were not included in the percentage calculation but were assigned as intermediate character in cluster analysis. All seabass isolates were identified to genus level as *Aeromonas* spp. considering the commercial kits used for identification.

#### 3.3.1. Electron Microscopy

**Figure 1:**
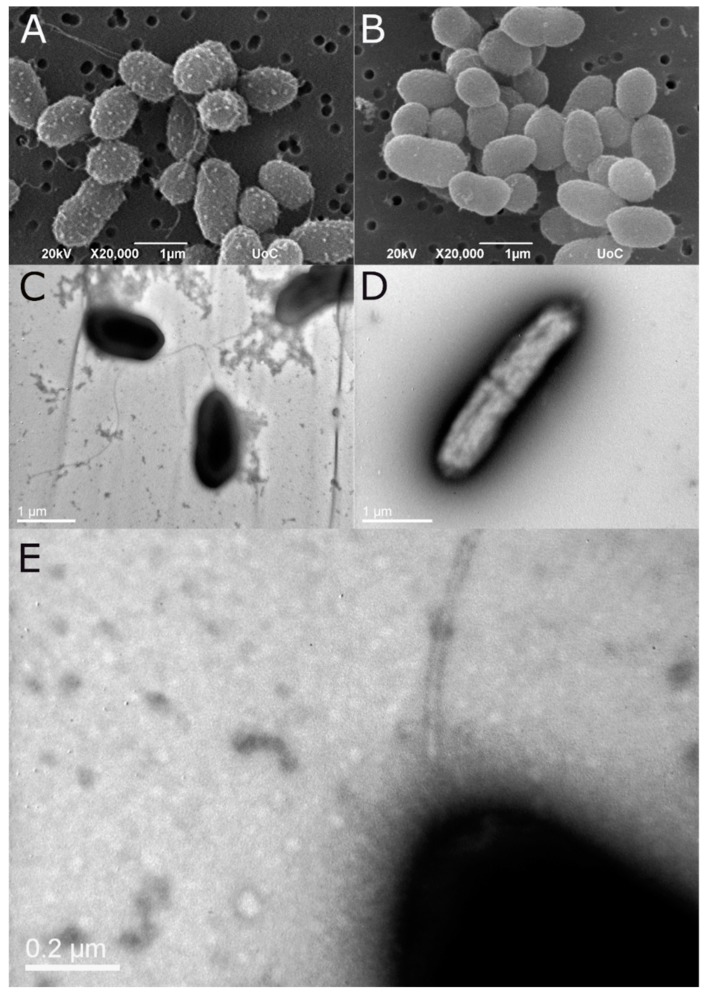
SEM micrograph of the motile strain PDB (**A**) and the non-motile NS (**B**). The same strains as shown in TEM following negative stain ((**C**,**D**), respectively). The presence of a polar flagellum is evident only in the motile strain (PDB). Higher magnification of the polar flagellum of strain PDB (**E**). Note the fuzzy coat of the cell possibly related to capsule.

Both TEM and SEM analysis confirmed that the non-motile strains lack flagella (Figure 1). On the contrary, motile strains possess a single polar flagellum. Lateral flagella were not observed in the examined samples. A fuzzy coat was observed around the cell of the motile strains both in TEM and SEM samples, a structure resembling bacterial capsule.

#### 3.3.2. Cluster Analysis

**Figure 2:**
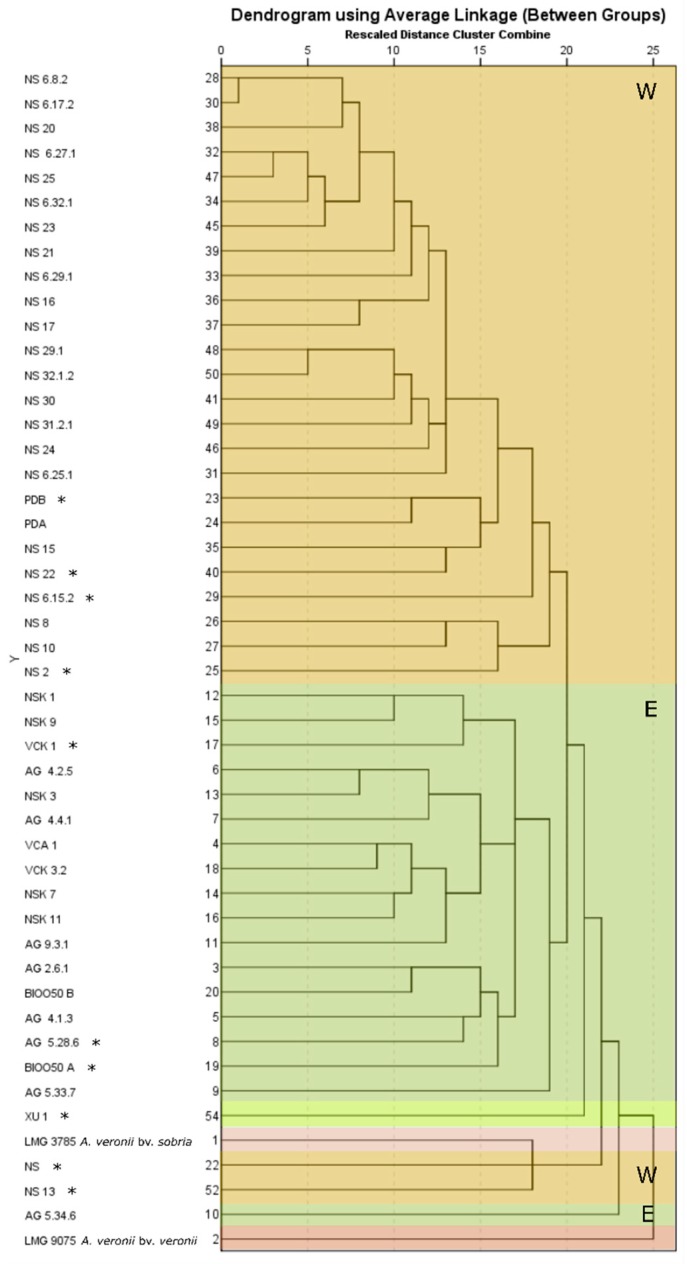
Dendrogram produced by Hierarchical cluster analysis on 45 A. veronii isolates from diseased seabass, the strains XU 1 and the type strains of A. veronii bv. sobria (LMG 3785) and A. veronii bv. veronii (LMG 9075). Characters analyzed included the API 20E, BIOLOG’s and catalase reactions, motility, pigment production, and β-hemolysis (119 characters). Western isolates are noted with W and eastern ones with E. Asterisk (*) indicates the sequenced strains’ genomes.

Five main groups were recognized in hierarchical cluster analysis (Figure 2): One consisting of the motile-pigment producing, West Aegean Sea isolates from Argolikos and Saronikos Bay, one consisting of the motile-(I)-pigment producing, East Aegean Sea isolates from Agathonisi Isl., Kalymnos Isl., and Turkey (Güllück), one represented by strain XU 1 from *X. helleri* alone, one consisting of the non-motile, non-pigment producing NS and NS 13 from West Aegean Sea grouped with the type strain of *A. veronii* bv. *sobria* (LMG 3785), and finally one represented by the most diverged among seabass isolates eastern strain AG 5.34.6 (sampling 18) which is motile-(I)-pigment producing. The type strain of *A. veronii* bv. *veronii* (LMG 9075) served as an outgroup in the present analysis. No internal group patterns were detected according to e.g., date of isolation or fish farm. When motility and pigment production were excluded from the dataset (dendrogram not presented), the group of NS, NS 13, and LMG 3785 (as well as the cluster of XU 1), were all clustered in the broader West Aegean Sea cluster representing the West/East geographic origin pattern for seabass isolates. Finally, the strains not included in the present analysis (NS 33.1, NS 49, NS 52, NS 58, T04-D, and Z 1) because of incomplete data on their biochemical properties, were analyzed separately including data of BIOLOG, catalase, motility, and pigment production. The strain Z 1 was grouped together with AG 5.34.6 and the rest with the West Aegean Sea group.

### 3.4. Antibiotic Susceptibility

Susceptibility test for the vibriostatic agent O/129, showed an inhibition zone (West Aegean isolates) or faint inhibition zone (East Aegean isolates) ranging between 7–9 mm for the majority of tested isolates. Nevertheless, growth was also evident in this area, or inhibition was not present after 48 h of incubation and thus, all isolates were finally assigned as resistant (Table 3) including the strains NS and PDB that were initially reported as sensitive [9]. The type strains LMG 3785 and LMG 9075 as well as the isolates XU 1 and Z 1 were also resistant to the vibriostatic agent O/129 (Table 3). All isolates were resistant to ampicillin. All seabass isolates were susceptible to oxytetracycline tetracycline, oxolinic acid, and florfenicol. All but one of them (NS 6.25.1) were susceptible to flumequine and all but two (NS 6.25.1 and NS 6.27.1) to Trimethoprim/sulfamethoxazole. Isolates XU 1 and Z 1 were resistant to oxytetracycline, tetracycline, and oxolinic acid. The Z 1 was also resistant to flumequine. Susceptibility to antibiotics agents is presented as mean inhibition diameter (mm) for each locality and as inhibition diameter for distinct strains (Table 4). Extreme values are presented separately.

### 3.5. Whole Genome Sequencing

Whole genome sequencing was performed on ten strains selected on the basis of geographic origin, fish farm and time of isolation, phenotypic characteristics, and host organism (Table 5). An overview of their genome features and Accession Numbers in NCBI GenBank are presented in Table S2. The de novo assembly resulted in 92 to 172 contigs with average length >27.5 kb and N_50_ values from 61.224,00 to 85.872,00. Full assembly data can be retrieved from Table S3. Quality data regarding the assembly following BUSCO analysis are shown in Table S4.

#### 3.5.1. Multilocus Sequence Typing (MLST)

**Figure 3:**
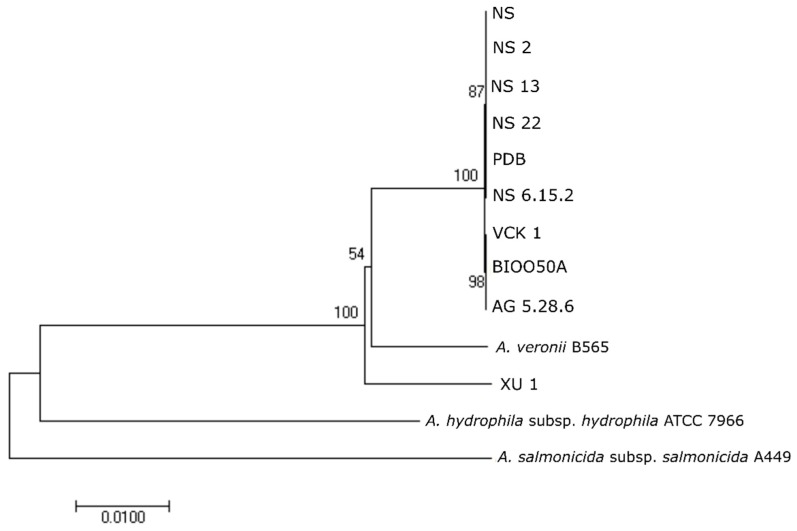
Phylogenetic relationships between seabass strains based on neighbor joining (NJ)-multilocus sequence typing (MLST) analysis on concatenated complete sequences of *gyr*B, *gro*L, *glt*A, *met*G, *pps*A, and *rec*A genes. Numbers on branches indicate the bootstrap values.

Comparison of the alleles of the nine *Aeromonas* strains from seabass in the PubMLST database, showed that all belonged to ST 23 (allele type for *gyr*B: 25, *gro*L: 24, *glt*A: 25, *met*G: 24, *pps*A: 22, and *rec*A: 24). This profile corresponded to the *A. veronii* strains Ae4 and Ae59, both isolated in 1999 from diseased European seabass from Italy. The seabass strains showed 100% similarity (0% genetic distance) in the complete gene sequences of *glt*A, *gro*L, *gyr*B, *met*G, and *rec*A while for *pps*A distance was calculated to 0.001%. Mean genetic distance in the *A. veronii* clade (seabass-XU 1-B565) was 0.006% for *glt*A, 0.011% for *gro*L, 0.006% for *gyr*B, 0.011% for *met*G, 0.019% for *pps*A, and 0.012% for *rec*A. In the NJ analyses, the nine seabass strains and XU 1 grouped together and formed a monophyletic clade with the *A. veronii* strain B565 (Figure 3). The strain XU 1 did not belong to any ST in the database.

#### 3.5.2. Whole Genome Comparison

**Figure 4:**
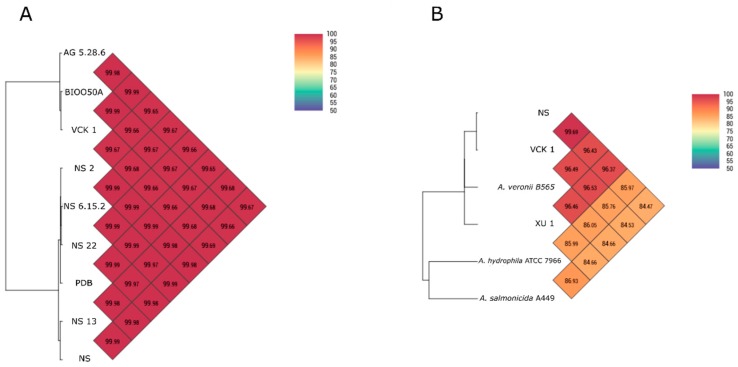
(**A**) Genome similarity Heatmap with OrthoANI values for the nine seabass strains sequenced. (**B**) Genome similarity Heatmap with OrthoANI values for the randomly selected representatives of West/East strains (NS, VCK 1) compared with other genomes of *Aeromonas* spp.

The nine seabass isolates were found closely related with calculated Ortho-ANI values ranging between 99.65%–99.99%. Isolates formed two groups (West/East Aegean Sea respectively) with internal Ortho-ANI similarity ≥99.97% (Figure 4A). Mean ANI values of strains NS and VCK 1 (randomly selected representatives for each geographic area) with 22 other *A. veronii* genomes were 96% ± 0.5% for both. Further comparisons with other *Aeromonas* spp. are presented in Figure 4B. All estimated ANI values are presented in Table S5.

**Figure 5:**
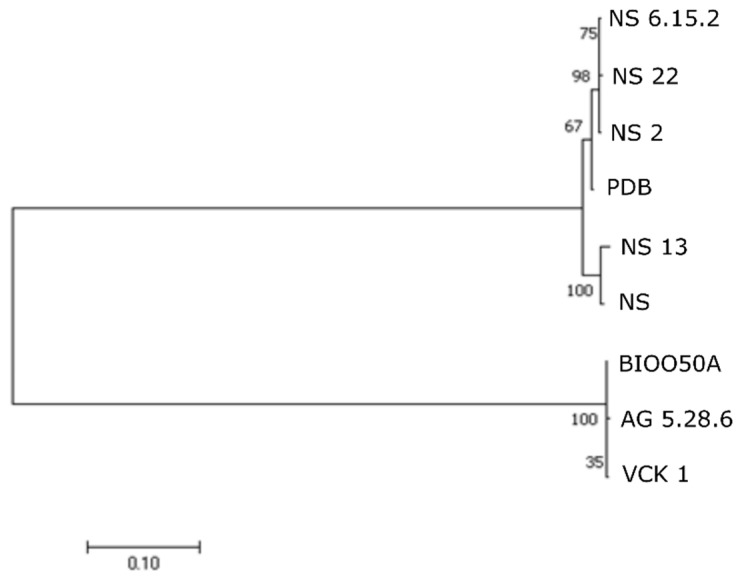
The evolutionary history of the nine strains from seabass inferred by maximum likelihood analysis on single nucleotide polymorphisms (SNPs).

The results of the SNPs analysis also grouped the isolates into two groups which represent their geographic origin (Figure 5). The average SNP difference among the eastern isolates was between 7–15 while the average SNP difference among the western isolates was between 6–168 (Table 6). Difference among western-eastern isolates was greater than 3700.

### 3.6. Outer Membrane Proteins’ Profile

In total 65 proteins were detected with PSORTb for outer membrane localization (score ≥8). Amongst them, 43 were predicted to have transmembrane (TM) beta-strands through PRED-TMBB2 and were further compared (Table S6). Eighteen porins were detected belonging to OmpA family (5), general bacterial porins (GBP) family (3), sugar porin family (2), outer membrane protein insertion porin (OmpIP/Omp85) family (2), FadL outer membrane protein (FadL) family (2), the outer membrane porins D (OprD) and OmpW, the poly-beta-1,6 N-acetyl-D-glucosamine export porin PgaA, and one hypothetical protein belonging to Nucleoside-specific Channel forming outer membrane porin (Tsx) family. Other outer membrane components detected include proteins of the outer membrane receptor (TonB) and outer membrane factor (TolC) families, the ShlB protein member of two-partner secretion (TPS) family, involved in the activation/secretion of hemolysin and the lipopolysaccharide assembly protein LptD. Secretins and filamentous hemagglutinin proteins were also detected.

The OMP profiles of the nine strains were very similar. All proteins retrieved from VCK 1 were detected in the other seabass genomes, and in most cases protein similarity was found ≥99%. In most cases the small differences found in the protein sequence reflected the West/East geographic origin of the seabass strains. Maltoporin LamB was distinct (53% similarity) among the West/East Aegean Sea isolates. The MshL protein sequence of strains PDB, NS 22, and AG 5.28.6 had stop codons, the BamA was different by 16–17 aa between West/East Aegean Sea isolates while the more recent western ones differed by 1 aa from the older ones. The transporter protein (transporter CDS-1) differed by 2 aa among the western non-motile, non-pigment-producing strains (NS, NS 13), the western motile, pigment producing (PDB, NS 2, NS 22, NS 6.15.2), and the eastern motile, I-pigment producing (VCK 1, BIOO50A, AG 5.28.6) strains.

The S-layer protein gene was found incomplete on strains NS, NS 2, PDB, VCK 1, and AG 5.28.6. Furthermore, S-layer protein sequences of strains BIOO50A (East) and NS 13, NS 22, and NS 6.15.2 (West) differed in length by 87 aa. Finally, the filamentous hemagglutinin N-terminal domain-containing protein was found to be frame-shifted including stop codons in strains PDB, VCK 1, and AG 5.28.6. The rest of the West/East Aegean Sea strains had identical protein sequences. Three tandem homologues of OmpA porin family were found: Porin OmpA CDS-2 (1020 bp): Porin OmpA CDS-1 (999 bp), and hypothetical protein CDS-8 (1038 bp).

Comparing with other strains, the aminoacidic sequences from seabass were generally more similar to the ones of strain B565. In a few cases such as TonB-dependent hemoglobin/transferrin/lactoferrin family receptor and the LPS-assembly protein LptD the opposite was observed. Moreover, the PgaA protein, ShlB/FhaC/HecB family hemolysin secretion/activation protein, S-layer family protein, and filamentous hemagglutinin were not detected neither in B565 nor XU 1 with the methodology followed here. Finally, the maltoporin LamB was identical among western isolates and strain B565.

### 3.7. Virulence

#### 3.7.1. Genomic Islands

All nine genomes contained GIs, with average 24 GIs per genome. From the GIs detected by the tools used, the ones greater than 9.5 kbp were chosen. The average length was 20,731 bp and the coverage of the genome approximately 10.4% (Table 7). The percentage of the hypothetical proteins within the GIs was the same with the genomes. Approximately 32% of the GIs contained virulence factors. As for the virulence genes in the GIs, the hemolysin D, RTX toxin, *Hcp1*, iron acquisition, type III secretion system, and flagellar proteins were detected. In addition, PDB and NS22 GIs contained a resistance-nodulation-cell division (RND) antibiotic efflux pump gene (*adeF*). According to the analysis of PHASTER the genomes of the nine strains had no intact prophage regions (PRs), but the number of the incomplete (completeness score less than 70) and questionable (completeness score 70–90) prophages varied from one to four (Table 7).

#### 3.7.2. Virulence Genes

The genes of virulence factors (secretion systems, flagella, toxins, etc.) that were detected manually and through PATRIC are listed in Table S7. Gene clusters of type II and type III secretion systems were present in all seabass strains (Table 8). Examining the type III secretion system, most of the genes composing the system’s cluster were found, and syntenies were conserved among the nine strains and other species of gram-negative bacteria according to the RAST annotation. Both apparatus’ proteins and effectors were included. The type VI secretion system gene cluster (about 21 kbp) was detected only on the eastern strains.

Great variability was found between strains in the exotoxins group (Table 9). The secreted proteins existed in multiple CDS in the genomes (Table S7), identical or not. The *Hcp1*, which is secreted by the type VI secretion system, was present in most of the strains. In BIOO50A was found at the end of the contig. The membrane fusion protein hemolysin D (*hlyD*) (type I secretion system), had seven CDS common between the seabass strains. The aerolysin family beta-barrel pore-forming toxin was complete only in the eastern strains, while in the western ones was detected fragmented by an integrase. Other virulence factors detected were the type IV pili twitching motility protein PilT, the MSHA biogenesis proteins, the TonB-ExbB-ExbD system, and iron acquisition proteins. The RGI predicted four antibiotic resistance genes, common in all the nine strains, the OXA beta-lactamase (*OXA-12*), the CphA beta-lactamase (*cphA3*), and two resistance-nodulation-cell division (RND) antibiotic efflux pump genes (*adeF*).

The *A. veronii* genomes had five gene clusters containing flagellar genes (Table 8 and Table S7). Seven more genes related to flagellum were detected scattered in other areas of the genome. Six of them encoded components of the flagellar motor (*motA*, *motB*, *motX*, *motY*, *pomA*, and *pomB*) and the last the *flgT*, whose protein is a component of H-ring (a structure required for the proper assembly of PomAB stator complex). Approximately 105 genes were detected involved in the formation of the flagellum. Following alignment of the respected loci and genes of the motile strain PDB and the non-motile NS, differences were identified in four genes out of the 105. These were the *flgG*, *flgT*, *fliI*, and *fliS*.

### 3.8. In Vivo Virulence Test

LD50 was calculated for both a 24 and 48 h period. The LD50-24 ranged from 4.2 × 10^5^ to 2.4 × 10^6^ and LD50-48 ranged from 3.3 × 10^5^ to 1.4 × 10^6^ cfu/fish (Table 10). After probit analysis and comparisons through the relative median potency table, the LD50 values were not found significantly different. There was a tendency for the non-motile western strains NS and NS13 strains to have the lowest LD50 values.

## 4. Discussion

*Aeromonas veronii* was isolated from diseased European seabass farmed in seven localities in both West and East Aegean Sea. The species was the most prevalent in all incidents of *Aeromonas*-suspected infections with key clinical signs lethargy, icteric appearance and petechial hemorrhages externally, splenomegaly and nodules on spleen and kidney internally, as initially described [9]. To our knowledge this is the first study after the description of the disease that directly correlates this pathology of seabass with *A. veronii* in a large geographic area. The study aimed to record and describe the variability of the pathogen in the affected areas and set the basis for the disease’s management in the future. Comparative analysis was conducted on different features of the pathogen.

*Aeromonas veronii* was the most prevalent species in all incidents of *Aeromonas* infections in seabass in both sides of the Aegean Sea (West/East). In the case of fish farm 1 (West) where the disease was initially described [9] we can now talk for an established problem as long as *A. veronii* was isolated from diseased fish almost all year round (March–December) excluding the winter months (temperature <18 °C), over the past ten years. Regarding the West Aegean Sea, while initially the disease was reported to affect only two neighboring fish farms in Argolikos Bay, it has now affected one more farm in the same area but more importantly it has geographically expanded to the neighboring Saronikos Bay where aquaculture activity is very intensive.

In the East Aegean Sea, the situation appeared less persistent. Reports for aeromonad outbreaks were not constant in time. Outbreaks also occurred during the summer months. Fish exhibiting the typical clinical signs of *A. veronii* disease are still present in the field in summer months (personal communications with fish farmers) but are not considered a threat for the moment because impacts are much lower than those recorded in the West Aegean Sea. Nevertheless, a recent outbreak in Bodrum (sampling 24) and the similarities of strain T04-D with the rest of eastern isolates should be considered when discussing the disease’s occurrence in East Aegean Sea. In any case, the ability to cause hemolysis in seabass blood agar was observed in all tested isolates, an attribute related to the hepatic jaundice and the icteric appearance of the diseased fish in the field but also to the in vivo hemolysis observed in seabass challenged with strains NS and PDB [9]. Pathogenicity tests in zebrafish showed similar virulence ability for all the strains. However, it should be noted that virulence in seabass is much higher following intraperitoneal injection with doses as low as 10^4^ cfu per fish resulting in 100% mortality within 48 h. This together with the fact that other fish species cultured in adjacent cages are not affected during disease outbreaks suggests either an adaptation of the pathogen to this host, or an increase sensitivity of seabass to *A. veronii*. This should be further investigated in future studies.

The same species (*A. veronii* bv. *sobria*) has been reported in diseased seabass and seabream in Italy [27], and from diseased seabass in one more case in the Black Sea [41]. In the latter, the bacterium was reported as the most prevalent species in diseased seabass, isolated alone, or in mixed-infections with *Ph. damselae* subsp. *damselae* and *Vibrio* spp. The sampling was conducted during summer and autumn in water temperature between 20–26 °C, in the same range with the outbreaks in the Aegean Sea. *A. veronii* has also been reported in diseased rainbow trout farmed in the Black Sea and in the Aegean Sea [42]. Fish transfers between farms located in the Black Sea and the Aegean regions could explain the establishment of the pathogen in the latter, however this should be confirmed in a future study using molecular epidemiological methods.

Three phenotypic groups can be described based on motility and pigment production: a) The West Aegean Sea, rarely isolated strains non-motile, non-pigment producing, b) the West Aegean Sea strains motile and pigment-producing, and c) the East Aegean Sea isolates motile and I-pigment-producing. Typical biochemical properties of *Aeromonas* spp., similar to positive catalase and oxidase reactions, inability to produce acid from xylose, absence of urease, and fermentation of D-glucose were evident for all isolates from seabass [43]. Production of acid from D-arabitol was evident for 47/50 isolates, only few grew in salinity over 4% NaCl, while trehalose fermentation was evident mainly for the western ones. Despite the lack of motility (NS and NS 13), pigment production (western isolates), and negative indole reaction (all seabass isolates) that are characteristics of *A. salmonicida* [44], all seabass isolates were negative for fermentation of L-arabinose and salicin which are characteristics of the *A. sobria* species complex [43]. In the *A. sobria* species complex, sucrose fermentation (all isolates) is characteristic for *A. veronii* and among the species’ biovarieties negative ODC reaction (all except for BIOO50A) is characteristic of *A. veronii* bv. *sobria* [43,45]. It is worth mentioning that indole negative *A. veronii* was reported in both cases of occurrence of the species in diseased seabass mentioned before; in Italy (strains Ae4 and Ae59) [27], and in the Black Sea [41]. Few pigment-producing isolates of *A. veronii* were also reported in [27]. The brown pigment produced by western strains seems to be pyomelanin which is produced by the tyrosine metabolism pathway [46]. The mechanism of pyomelanin production as well as its importance as competitive advantage [47] for *A. veronii* is the subject of a parallel study of our group (manuscript in preparation).

Biochemically, the only common characteristic among seabass strains was the negative indole reaction which was also evident in other studies [27,41]. Whether this is an adaptation that gives advantage to these *A. veronii* strains compared to other aeromonads or other common marine pathogens, has to be investigated as long as indole has been correlated to key aspects of bacterial physiology like drug resistance, biofilm formation, and virulence [48]. For example, non-indole-producing bacteria use diverse oxygenases, which degrade indole from other species producing derivatives implicated in bacterial competition [48].

The resistance to ampicillin (penicillins) together with first generation cephalosporins is genetically encoded in aeromonads [49]. Two beta-lactamase genes were found in the genomes that could justify the ampicillin-resistant phenotype. However, the seabass strains studied here were susceptible to all the other registered for use in aquaculture antibiotics tested. Acquired resistance to antibiotics, e.g., tetracycline, oxolinic acid and flumequine has been reported in aeromonads in aquaculture systems [50,51]. Thus, one assumption would be that the multiple susceptibility detected here for *A. veronii* indicates a relatively recent appearance of the species in the Aegean Sea or a more rationale use of antibiotics according to the best practices in the Greek aquaculture industry. In any case, the species has been reported in seabass approximately in the last decade.

Regardless of the phenotypic divergence and the West/East pattern of biochemical diversification shown in cluster analysis, the western-eastern seabass isolates were almost indistinguishable by the majority of the genetic markers used for identification. This was also seen in the phylogenetic analyses in which the strain Ae4 isolated from diseased seabass in Italy showed 0% divergence from the seabass isolates in the Aegean Sea while strain, e.g., XU 1 from *X. helleri* was distinct. At the genomic level, high genomic similarity was observed as shown from Ortho-ANI values. The SNPs analysis was consistent with the patterns of phenotypic and geographic origin diversification. Despite the difference in isolation time, the non-motile, non-pigment producing western strains were closer to each other compared with the motile, pigment-producing ones. The same was evident also for the eastern strains (motile, non-pigment producing). The West/East pattern was clearly demonstrated as the difference among strains of different areas that was at least one order of magnitude higher than among strains of the same area. Divergence among *A. veronii* seabass isolates were also at least one order of magnitude higher than the ones reported in *A. salmonicida* subsp. *salmonicida* isolated from fresh and seawater farms of trout in Denmark which had a time gap of isolation of approximately 30 years [30].

While for *A. salmonicida,* host related homogeneity among subpopulations of strains has been proposed in various cases using different methodologies [52], the situation described for *A. veronii* here might be more complicated as also reported elsewhere [53]. Despite the fact that the particular strains of the species affected only seabass and not seabream nor other species farmed in neighboring cages [9], zebrafish was a successful model for the pathogenicity assay as long as mortality reached 100% within 48 h post challenge, at higher, however, doses from the respective needed for seabass [9]. High genomic similarity as observed here has been reported for virulent strains of *A. hydrophila* of the same ST which showed 99.81%–100% similarity in ANI values despite their distant geographic origin (USA, China), isolation source (fish and soil) and time of isolation (1989–2010) [54]. Furthermore, the divergence among western-eastern isolates through SNPs analysis is suggestive of independent evolutionary routes of the pathogen in the two sides of the Aegean Sea at least for the last decade that have been detected and isolated. Whether there is a common indole negative ancestor, or this is a case of convergent evolution with adaptive advantage for the pathogen to a new environment, e.g., marine environment, needs further investigation. It should be noted that pathogenic mesophilic aeromonads are mostly found in environments of lower salinity. Additionally, the host range of the pathogen is not defined yet and host physiology should be taken under consideration for possible vulnerability to bacterial pathogens or groups of pathogenic taxa.

The outer membrane proteins were studied as long as they are related to virulence and pathogenicity involving in the attachment and invasion of bacteria to the host. Many known OMPs have been tested as vaccine candidates as they have strong immunogenic effects. The outer membrane protein W has been shown to be highly conserved among *Aeromonas* spp. but has also homology with the OmpW of other taxa [55]. Common carp (*Cyprinus carpio*) intraperitoneally vaccinated and rohu (*Labeo rohita*) orally vaccinated with recombinant OmpW from *A. hydrophila* produced antibody titers and showed higher relative percent survival (RPS%) compared to the control groups following challenge tests [56,57]. Recombinant protein OmpC (general porin family), from *A. hydrophila* induced immune response in mice [58]. The porin CDS-2 from strain VCK 1 showed 84% similarity in gene sequence (HF546053) and 87% similarity with protein OmpC of *A. hydrophila* (EUS112) [58]. Both OmpW and OmpC were highly conserved among the seabass isolates and therefore could be considered as good candidates for vaccine development.

Other highly conserved membrane proteins among seabass isolates were the porins OmpA which are members of the well-studied OmpA protein family with key role in bacterial pathogenicity [59]. In *A. veronii* from the intestinal tract of carp, two tandem homologues (OmpAI and OmpAII) have been reported and the proteins were related to adhesion of the bacterium to the surface of the intestinal tract of common carp [60]. Here, these homologues corresponded to OmpA porins CDS-1 and CDS-2 and were found tandem with a third homologue (hypothetical CDS-8) with total protein similarity in this group of three homologues >50%. Comparison with sequences reported from carp (AB290200), OmpAI showed 92% similarity to OmpA porin-2 while OmpAII 99% similarity to OmpA porin-1 from seabass. This was the highest similarity of OmpAI and OmpAII from carp when comparing to the other seabass homologues of OmpA-porins. An oral recombinant vaccine with *Lactobacillus casei* expressing the OmpAI induced immune response and protection against challenge with *A. veronii* in common carp [61].

Few outer membrane proteins varied significantly among seabass isolates in the two sides of the Aegean like maltoporin LamB and S-layer. Both of these membrane substances have been studied and are capable to induce immune response and protection in fish [62,63,64]. Whether these differences are significant in the development of an *A. veronii* vaccine for seabass has to be further investigated. In terms of strain selection for the development of a bacterin vaccine for the Aegean Sea, a bivalent preparation containing eastern and western strains would be worth trying. Protein distances among seabass strains and strains B565 and XU 1 indicated that at least some proteins were highly conserved among them. This should be further investigated including more *A. veronii* genomes from different hosts and isolation sources in order to detect for instance possible host-related similarities or highly conserved proteins that could serve as antigens for recombinant vaccines for *A. veronii*.

The coverage of the GIs in the genomes of the seabass strains (>10%) laid in the higher part of the range described for marine bacteria (3%–12%) [65] where in the 50% of the studied genomes the GI coverage was approximately 3%. Mobile elements are important for bacterial evolution as they often hand in virulence factors and fitness features. Preliminary analysis detected virulence factors similar to the genes of the type III secretion system in the seabass’ GIs as described elsewhere [66]. In addition, the analogy of prophages per genome detected in the current study, although not intact, is mentioned before in another species of the genus, *A hydrophila* [54,67,68]. Prophage genes are widely distributed within the aeromonads [69,70], but in the course of evolution there is a constant process of losing and acquiring such genetic material. Further analysis is needed in order to study the relationship among GIs’ size and composition and prophage occurrence to the pathogenicity of the *A. veronii* studied herein.

One of our aims was to clarify if the phenotypic variability of the strains that is the presence and absence of the polar flagellum imposed a difference in the virulence of the bacteria. The non-motile strains had slightly lower LD50, when intraperitoneally injected, which can be due to the fact that the lack of flagella favors them. The flagella are major antigenic elements and the presence of both flagellated and non-flagellated bacteria within a species and also the switch from the one state to the other happens to pathogens and environmental isolates [71,72]. The flagella are strictly regulated structures and they are not formed or do not function if one of the motility genes is missing [73,74] or is non-functional [75]. Between the four genes that differed among the strains, the *flgT* is more important for the stability and the secretion of the flagellum. The mutants of *A. hydrophila* with *flgT* gene deleted had no flagellum [76] contrary to the deletion of the *fliS* that resulted in phenotypes with reduced motility in *Y. pseudotuberculosis* [77].

The type II and III secretion systems, prominent structures for the protein transport through the cell envelope, were conserved in all nine strains. There were three clusters of genes of the T3SS and one of the T2SS, containing the components of the apparatuses. The T3SS is considered key factor for the infection mechanism of *A veronii* bv. *sobria* [78]. Its function is linked to the resistance of the bacteria to the immune response of the host [79]. In addition, the T2SS participates in the translocation of toxins and assemblage of the type IV pili in the outer membrane [80]. The type VI secretion system was not detected in the strains of West Aegean Sea and the lack of the cluster was a considerable difference. The system is significant for intrabacterial communication and competition but it is also a dominance mechanism within the gut environment [81,82,83]. However, its absence has been reported again in pathogens of the genus [54,84] and in *Bacteroides fragilis* the presence of the system varied among strains occupying the same niche [81], which is similar to our case. To sum up, it seems that the first two functional secretion systems are more crucial than the lack of the latter, considering that the strains caused similar pathogenicity in the in vivo studies. 

Other genes detected are a set of common exotoxins for aeromonads, including hemolytic enzymes, and iron-binding and transport genes. The *A. veronii* strains exhibit intense hemolytic activity and they are adapted to fish blood causing greater hemolytic effect than in mammal blood, as shown in the previous study [9]. There are many related genes inbuilt in the genome of the strains similar to hemolysins, hemagglutins, the TonB-ExbB-ExbD iron-binding transport system, and iron uptake regulation genes such as the FUR transcriptional factor that justify their virulence. An important difference in the strains of West Aegean Sea compared to the East Aegean ones, is that they have two fragments of aerolysin family toxin due to the presence of an integrase. Aerolysin is responsible for the total osmotic lysis of blood cells and is a major virulence factor of the species. In a recent study of *A. veronii,* the deletion of aerolysin resulted in a rapid loss of virulence of the pathogen and increased survival of the challenged animals [6]. In our study, in vivo virulence using zebrafish as a model is similar, however results from the field suggest that the West Aegean Sea isolates cause significantly higher mortalities to the cultured seabass.

## 5. Conclusions

The results of this study support the existence of a genetically distinct group of *Aeromonas veronii* bv. *sobria* strains that causes pathology to farmed European seabass. The bacteria carry most of the known virulence factors that are implicated in the pathogenicity. The spread of the disease observed over the past years in different fish farms in Greece and Turkey is alarming and since the use of antibiotics is being questioned in connection to consumer’s safety and environmental impacts, the development of an efficacious vaccine is in urgent need. Towards this direction, the genomic information related to antigenic proteins presented here can establish the basis for a future study.

## Figures and Tables

**Table 1:** The number of samplings conducted, the locations and dates of samplings, and the fish species sampled.

# Sampling	Area	Locality	Fish Farm	Fish Species	Sampling Date
1 *	West Aegean Sea	Argolikos Bay	1–2	*D. labrax*	2009 [9]
2–3		Argolikos Bay	1–2		9/2015
4		Argolikos Bay	1–2		11/2015
5		Argolikos Bay	1–2		3/2016
6–7		Argolikos Bay	1–2		9/2016
8 *		Argolikos Bay	1–2		6/2018
9		Argolikos Bay	1–2		9/2018
10		Argolikos Bay	1–2		10/2018
11		Argolikos Bay	3		12/2015
12		Saronikos Bay	4		9/2016
13 *		Saronikos Bay	4		5/2018
14 *		Saronikos Bay	4		6/2018
15	East Aegean Sea	Agathonisi Isl.	5		4/2015
16 *		Agathonisi Isl.	5		6/2015
17		Agathonisi Isl.	5		9/2015
18		Agathonisi Isl.	5		11/2015
19 **		Agathonisi Isl.	5		7/2016
20 *		Kalymnos Isl.	6		6/2015
21–22		Kalymnos Isl.	6		9/2015
23 *		Güllück	7		2010
24 *		Bodrum	8		3/2019
25 *	Other	N/E Greece	9	*X. helleri*	2/2015
26 *		Crete	10	*D. rerio*	12/2018

*: Outbreak, **: transportation

**Table 2:** Number of seabass examined per sampling, *Aeromonas* spp. detection and prevalence on diseased fish per sampling, number of *Aeromonas* spp. isolates analyzed in the present study, and the isolates’ codes.

Area	# Sampling	# Examined Fish	Prevalence in Diseased Fish	# Isolates Analyzed Herein	Isolate Code
**West Aegean Sea**	1	[9]	[9]	3	NS, PDA, PDB
	2–3	≥7		3	NS 2, 8, 10 [9]
	4	33	33% (11/33)	7	NS 6 [9]
	5	≥5		3	NS 15–17
	6–7	≥3		3	NS 20–22
	8	≥1		1	NS 30
	9	≥1		1	NS 49
	10	≥9		2	NS 52–58
	11	≥1		1	NS 13
	12	≥3		3	NS 23–25
	13	11	73% (8/11)	2	NS 29, 31
	14	≥10		2	NS 32–33
SUM West				31	
**East Aegean Sea**	15	13	15% (2/13)	3	AG 2
	16	≥1		1	VCA
	17	4	75% (3/4)	4	AG 4
	18	26	11% (3/26)	3	AG 5
	19	5	20% (1/5)	1	AG 9
	20	≥8		3	NSK 1–8
	21–22	≥9		4	NSK 9–12, VCK 1–5
	23	≥2		2	BIOO50A, B
	24	≥1		1	T04-D
SUM East				22	
**Other**	25			1	XU 1
	26			1	Z 1
SUM				55	

**Table 3:** Results on API 20E reactions, catalase, β-hemolysis, motility, and pigment production presented as positive/negative reactions for the type strains of *A. veronii* (LMG 3785 and LMG 9075), strains XU 1 and Z 1, and as a percentage of positive reactions for each area (West/East) for isolates from seabass.

Reaction/Strain	a	b	c	d	West Aegean Sea (%)	East Aegean Sea (%)
**ONPG**	+	+	+	ND	15 (4/27)	89 (16/18)
**ADH**	+	−	+	ND	100	44 (8/18)
**LDC**	+	+	+	ND	96 (26/27)	28 (5/18)
**ODC**	−	+	−	ND	0	6 (1/18)
**CIT**	−	+	+	ND	85 (23/27)	67 (12/18)
**H2S**	−	−	−	ND	0	0
**URE**	−	−	−	ND	0	0
**TDA**	+	+	+	ND	96 (26/27)	100
**IND**	+	+	+	ND	0	0
**VP**	+	+	−	ND	26 (7/27)	50 (9/18)
**GEL**	+	+	+	ND	81 (22/27)	100
**GLU**	+	+	+	ND	100	100
**MAN**	+	+	+	ND	100	100
**INO**	−	−	−	ND	0	0
**SOR**	−	−	−	ND	0	0
**RHA**	−	−	−	ND	0	0
**SAC**	+	+	+	ND	100	100
**MEL**	−	−	−	ND	0	0
**AMY**	−	−	−	ND	0	0
**ARA**	−	−	−	ND	0	0
**OX**	+	+	+	ND	100	100
**Catalase**	+	+	+	+	100 (31/31)	100 (19/19)
**β-hemolysis**	+	+	+	ND	100 (31/31)	100 (18/18)
**Motility**	+	+	+	+	94 (29/31)	100 (19/19)
**Pigment production**	−	−	−	−	94 (29/31)	100 (19/19) *
**O/129**	+	+	+	+	100 (22/22)	100 (15/15)

a: LMG 3785 *A. veronii* bv. *sobria*, b: LMG 9075 *A. veronii* bv. *veronii*, c: XU 1, d: Z 1, (+): Positive reaction, (−): Negative reaction, ND: Not done, * Intermediate pigment production.

**Table 4:** Antibiotic susceptibility profiles for *Aeromonas* spp. isolates presented as mean inhibition diameter (mm) for the isolates of each locality and as inhibition diameter for unique ones. Outliers are presented below the mean diameter.

# Sampling	Area	Locality	# Isolates/Locality	Inhibition Diameter						
				OT (30 μg)	TE (30 μg)	UB (30 μg)	SXT (25 μg)	OA (2 μg)	FFC (30 μg)	AMP (10 μg)
1–10	W	Argolikos Bay	22	37 ± 3	36 ± 4	35 ± 5	28 ± 7	33 ± 3	39 ± 3	0
			(NS 6.25.1)			19	11			
			(NS 6.27.1)				6			
11	W	Argolikos Bay	1 (NS 13)	42	42	44	30	38	42	
12–14	W	Saronikos Bay	7	37 ± 2	37 ± 2	35 ± 3	28 ± 3	32 ± 3	39 ± 2	0
15–19	E	Agathonisi Isl.	9	37 ± 1	36 ± 2	37 ± 1	22 ± 2	31 ± 1	40 ± 1	0
20–22	E	Kalymnos Isl.	7	35 ± 1	36 ± 2	36 ± 1	20 ± 3	30 ± 2	38 ± 2	0
23	E	Güllück	2	35 ± 4	35 ± 1	35 ± 5	21 ± 1	34 ± 0	41 ± 1	0
24	E	Bodrum	1 (T04-D)	30	30	33	22	29	37	0
25	other	N/E Greece	1 (Z 1)	7	11	0	24	0	34	0
26	other	Crete	1 (XU 1)	7	12	28	24	15	40	0

W: West Aegean Sea, E: East Aegean Sea.

**Table 5:** Characteristics of the selected for whole genome sequencing strains.

Strain	Area	Locality (Fish Farm)	Host	Collection Date	Motility	Pigment
**NS**	W	Argolikos Bay (1–2)	*D. labrax*	2009 [9]	−	−
**PDB**	W	Argolikos Bay (1–2)	*D. labrax*	2009 [9]	+	+
**NS 2**	W	Argolikos Bay (1–2)	*D. labrax*	2015	+	+
**NS 6.15.2**	W	Argolikos Bay (1–2)	*D. labrax*	2015	+	+
**NS 13**	W	Argolikos Bay (3)	*D. labrax*	2015	−	−
**NS 22**	W	Argolikos Bay (1–2)	*D. labrax*	2016	+	+
**AG 5.28.6**	E	Agathonisi Isl. (5)	*D. labrax*	2015	+	I
**VCK 1**	E	Kalymnos Isl. (6)	*D. labrax*	2015	+	I
**BIOO50A**	E	Güllück (7)	*D. labrax*	2010	+	I
**XU 1**	other	N/E Greece (9)	*X. helleri*	2015	+	−

W: West Aegean Sea, E: East Aegean Sea, (+): Positive reaction, (−): Negative reaction, I: Intermediate pigment production.

**Table 6:** Single nucleotide polymorphisms (SNPs) matrix of the nine sequenced *A. veronii* isolates.

	NS	PDB	NS 2	NS 6.15.2	NS 13	NS 22	AG 5.28.6	VCK 1	BIOO50A
**NS**									
**PDB**	119								
**NS 2**	146	49							
**NS 6.15.2**	137	40	11						
**NS 13**	40	139	168	159					
**NS 22**	139	40	15	6	159				
**AG 5.28.6**	3795	3836	3863	3854	3817	3856			
**VCK 1**	3797	3838	3856	3856	3819	3858	18		
**BIOO50A**	3794	3835	3862	3853	3816	3855	15	7	

**Table 7:** Main features of the genomic islands and prophage regions of the nine seabass strains.

Strain	Num. of GIs (>9.5 kbp)	Num. of Pathogenicity Islands	Mean Length (bp)	Total Size (bp)	Total Size of GIs/Genome Size	Num. HP/CDS in GIs	Num. of Incomplete PRs	Num. of Questionable PRs	Mean Length of PRs (bp)
**NS**	22	10	21,734.45	478,158	0.10	0.44	1	0	5800
**PDB**	27	11	19,939.22	538,359	0.11	0.46	3	1	11,850
**NS 2**	22	7	22,938.23	504,641	0.11	0.54	4	1	7500
**NS 6.15.2**	23	7	19,148.00	440,404	0.09	0.51	1	0	34,100
**NS 13**	29	12	21,208.76	615,054	0.13	0.51	2	0	5700
**NS 22**	29	10	20,771.86	602,384	0.13	0.53	1	1	8900
**AG 5.28.6**	21	5	20,606.10	432,728	0.09	0.49	4	0	12,825
**VCK 1**	19	5	21,704.58	412,387	0.09	0.53	2	0	6100
**BIOO50 A**	19	6	18,523.74	351,951	0.08	0.55	3	1	11,850

GIs: Genomic islands, HP: Hypothetical proteins, PRs: Prophage regions.

**Table 8:** Prominent virulence factors’ gene clusters detected in seabass strains.

Virulence Factors	Gene Cluster	West Aegean	East Aegean
Type II secretion system	Cluster 1	+	+
Type III secretion system	Cluster 1	+	+
	Cluster 2	+	+
	Cluster 3	+	+
Type VI secretion system	Cluster 1	−	+
Flagellar proteins	Cluster 1	+	+ ^a^
	Cluster 2	+	+ ^b^
	Cluster 3	+	+
	Cluster 4	+	+
	Cluster flaggelins	+ ^c^	+

(+): detected, (−): not detected, a: Incomplete sequence in VCK 1, b: Incomplete sequence in B, IOO50A, c: Incomplete sequence in NS and NS 13.

**Table 9:** Secreted proteins/exotoxins detected in seabass strains.

Virulence Factors	West Aegean	East Aegean	% Identity
Hcp1	+	+ ^a^	>90
hemolysin secretion protein D (1.419 bp)	+	+	>99
Hemolysin III	+	+	>99
Aerolysin family beta-barrel pore-forming	− ^b^	+	<60
Hemagglutinin	+	+	>99
RTX toxin	+	+	>99

(+): detected, (−): not detected, a: Incomplete sequence, b: Fragmented sequence.

**Table 10:** Probit analysis LD50 (cfu/fish) of the nine seabass strains at 24 and 48 h.

Strain	LD50 (24 h)	95% Confidence Interval	LD50 (48 h)	95% Confidence Interval
**NS**	4.2 × 10^5^	(2.6 × 10^4^, 4.5 × 10^6^)	3.3 × 10^5^	(1.4 × 10^4^, 4.3 × 10^6^)
**PDB**	2.4 × 10^6^	(1.2 × 10^5^, 2.3 × 10^7^)	1.4 × 10^6^	(4 × 10^4^, 1.7 × 10^7^)
**NS 2**	1.6 × 10^6^	(1.3 × 10^5^, 1.3 × 10^7^)	1.1 × 10^6^	(5.8 × 10^4^, 10^7^)
**NS 6.15.2**	1.1 × 10^6^	(8.6 × 10^4^, 1.1 × 10^7^)	5.4 × 10^5^	(8.5 × 10^3^, 7.2 × 10^6^)
**NS 13**	5.4 × 10^5^	(3.6 × 10^4^, 6.6 × 10^6^)	3.3 × 10^5^	(1.4 × 10^4^, 4.9 × 10^7^)
**NS 22**	10^6^	(7.3 × 10^4^, 10^7^)	8.4 × 10^5^	(3.9 × 10^4^, 10^7^)
**AG 5.28.6**	7.1 × 10^5^	(1.1 × 10^4^, 10^7^)	6.9 × 10^5^	(7.1 × 10^3^, 10^7^)
**VCK 1**	7.9 × 10^5^	(5.1 × 10^4^, 9.6 × 10^6^)	7.8 × 10^5^	(3.6 × 10^4^, 1.1 × 10^7^)
**BIOO50 A**	1.3 × 10^6^	(5.8 × 10^4^, 1.2 × 10^7^)	10^6^	(3.7 × 10^4^, 1.1 × 10^7^)

## Supplementary Materials

The following are available online at https://www.mdpi.com/2076-2607/7/11/504/s1. Figure S1: Phylogenetic relationships between strains from seabass and other *Aeromonas* spp. according to the topology of NJ analysis for *gyr*B gene; Table S1: Results on BIOLOG GEN III Microplate; Table S2: Overview of the sequenced strains and their corresponding genome features; Table S3: Assembly statistics of the sequenced strains; Table S4: Assessment of genome assemblies using BUSCO; Table S5: Similarity of strains NS and VCK 1 with other *A. veronii* strains and *Aeromonas* species expressed as % ANI values; Table S6: The outer membrane proteins of seabass strains; Table S7: Virulence factors of seabass strains.
