# Supplementary material for: Comparative Study on A Novel Pathogen of European Seabass. Diversity of Aeromonas veronii in the Aegean Sea
Source: Microorganisms. 2019 Oct 29;7(11):504. doi: 10.3390/microorganisms7110504 (PMC6921072; doi:10.3390/microorganisms7110504)
Supplement: Supplementary file 1 [file microorganisms-07-00504-s001.zip › Tables-Supplementary.docx]

**Table S1.** Results on BIOLOG GEN III Microplate reactions presented as positive/negative reactions for the type strains of *A. veronii* (LMG 3785 and LMG 9075), strains XU 1 and Z 1 and as a percentage of positive reactions for each area (West/East) for isolates from seabass.

| **#** | **Substrate** | **a** | **b** | **c** | **d** | **W (%)** | **E (%)** | **#** | **Substrate** | **a** | **b** | **c** | **d** | **W (%)** | **E (%)** |
| --- | --- | --- | --- | --- | --- | --- | --- | --- | --- | --- | --- | --- | --- | --- | --- |
| **A1** | Negative control | - | - | - | - | 0 | 0 | **E1** | Gelatin | + | + | - | + | 6 (2/31) | 16 (3/19) |
| **A2** | Dextrin | + | + | + | + | 100 | 100 | **E2** | Glycyl-L-Proline | + | I | + | + | 97 (30/31) | 100 |
| **A3** | D-Maltose | + | + | + | + | 100 | 100 | **E3** | L-Alanine | + | + | I | + | 90 (28/31) | 79 (15/19) |
| **A4** | D-Trehalose | + | + | + | + | 100 | 11 (2/19) | **E4** | L-Arginine | + | - | I | + | 97 (30/31) | 58 (11/19) |
| **A5** | D-Cellobiose | - | + | - | + | 0 | 0 | **E5** | L-Aspartic Acid | + | + | + | + | 97 (30/31) | 95 (18/19) |
| **A6** | Gentiobiose | - | - | - | - | 0 | 0 | **E6** | L-Glutamic Acid | + | + | + | + | 97 (30/31) | 95 (18/19) |
| **A7** | Sucrose | + | + | + | + | 100 | 100 | **E7** | L-Histidine | + | + | + | + | 94 (29/31) | 100 |
| **A8** | D-Turanose | - | + | - | - | 0 | 0 | **E8** | L-Pyroglutamic Acid | - | - | - | - | 0 | 0 |
| **A9** | Stachyose | - | - | - | - | 0 | 0 | **E9** | L-Serine | + | + | + | + | 100 | 100 |
| **A10** | Positive control | + | + | + | + | 100 | 100 | **E10** | Lincomycin | + | + | - | + | 87 (27/31) | 100 |
| **A11** | pH 6 | + | + | + | + | 100 | 100 | **E11** | Guanidine HCl | + | + | + | + | 100 | 100 |
| **A12** | pH 5 | - | - | + | + | 55 (17/31) | 42 (8/19) | **E12** | Niaproof 4 | + | + | + | + | 100 | 100 |
| **B1** | D-Raffinose | - | - | - | - | 0 | 0 | **F1** | Pectin | + | + | + | I | 35 (11/31) | 21 (4/19) |
| **B2** | α-D-Lactose | - | - | - | - | 0 | 0 | **F2** | D-Galacturonic Acid | - | - | - | - | 0 | 0 |
| **B3** | D-Melibiose | - | - | - | - | 0 | 0 | **F3** | L-Galactonic Acid Lactone | - | - | - | - | 0 | 0 |
| **B4** | β-Methyl-D-Glucoside | - | + | + | + | 0 | 100 | **F4** | D-Gluconic Acid | + | + | + | + | 100 | 16 (3/19) |
| **B5** | D-Salicin | - | + | - | - | 0 | 5 (1/19) | **F5** | D-Glucuronic Acid | - | - | - | - | 0 | 0 |
| **B6** | N-Acetyl-D-Glucosamine | + | + | + | + | 100 | 95 (18/19) | **F6** | Glucuronamide | - | - | - | - | 3 (1/31) | 16 (3/19) |
| **B7** | N-Acetyl-β-D-Mannosamine | - | - | - | - | 3 (1/31) | 0 | **F7** | Mucic Acid | - | - | - | - | 0 | 0 |
| **B8** | N-Acetyl-D-Galactosamine | + | + | + | - | 94 (29/31) | 95 (18/19) | **F8** | Quinic Acid | - | - | - | - | 3 (1/31) | 0 |
| **B9** | N-Acetyl Neuraminic Acid | - | - | - | - | 0 | 0 | **F9** | D-Saccharic Acid | - | - | - | - | 0 | 0 |
| **B10** | 1 NaCl | + | + | + | + | 100 | 100 | **F10** | Vancomycin | + | + | + | + | 100 | 100 |
| **B11** | 4 NaCl | - | - | - | + | 6 (2/31) | 21 (4/19) | **F11** | Tetrazolium Violet | - | + | - | + | 55 (17/31) | 47 (9/19) |
| **B12** | 8 NaCl | - | - | - | - | 0 | 0 | **F12** | Tetrazolium Blue | + | + | + | + | 90 (28/31) | 95 (18/19) |
| **C1** | α-D-Glucose | + | + | + | + | 100 | 100 | **G1** | p-Hydroxy- Phenylacetic Acid | - | - | - | - | 0 | 0 |
| **C2** | D-Mannose | + | + | + | + | 100 | 95 (18/19) | **G2** | Methyl Pyruvate | + | + | + | + | 87 (27/31) | 32 (6/19) |
| **C3** | D-Fructose | + | + | + | + | 100 | 100 | **G3** | D-Lactic Acid Methyl Ester | - | - | - | - | 0 | 0 |
| **C4** | D-Galactose | + | + | + | + | 97 (30/31) | 95 (18/19) | **G4** | L-Lactic Acid | - | - | - | - | 0 | 5 (1/19) |
| **C5** | 3-Methyl Glucose | - | - | - | - | 0 | 5 (1/19) | **G5** | Citric Acid | - | + | + | + | 19 (6/31) | 0 |
| **C6** | D-Fucose | - | I | - | + | 3 (1/31) | 5 (1/19) | **G6** | α-Keto-Glutaric Acid | - | - | - | - | 0 | 0 |
| **C7** | L-Fucose | - | - | - | - | 0 | 0 | **G7** | D-Malic Acid | - | - | - | - | 0 | 0 |
| **C8** | L-Rhamnose | - | - | - | - | 0 | 0 | **G8** | L-Malic Acid | + | + | + | + | 100 | 100 |
| **C9** | Inosine | + | + | + | + | 100 | 100 | **G9** | Bromo-Succinic Acid | + | I | + | - | 0 | 5 (1/19) |
| **C10** | 1 Sodium Lactate | + | + | + | + | 100 | 100 | **G10** | Nalidixic Acid | - | - | + | + | 0 | 0 |
| **C11** | Fusidic Acid | - | - | - | - | 0 | 11 (2/19) | **G11** | Lithium Chloride | - | + | - | - | 0 | 0 |
| **C12** | D-Serine | + | + | + | + | 100 | 95 (18/19) | **G12** | Potassium Tellurite | - | - | - | - | 3 (1/31) | 0 |
| **D1** | D-Sorbitol | - | - | - | - | 0 | 0 | **H1** | Tween 40 | + | - | + | + | 97 (30/31) | 79 (15/19) |
| **D2** | D-Mannitol | + | + | + | + | 100 | 100 | **H2** | γ-Amino-Butryric Acid | - | - | - | - | 0 | 0 |
| **D3** | D-Arabitol | - | - | - | - | 6 (2/31) | 5 (1/19) | **H3** | α-Hydroxy- Butyric Acid | - | - | - | - | 0 | 0 |
| **D4** | myo-Inositol | - | - | - | - | 0 | 0 | **H4** | β-Hydroxy-D,L-Butyric Acid | - | - | - | - | 0 | 0 |
| **D5** | Glycerol | + | + | + | + | 97 (30/31) | 100 | **H5** | α-Keto-Butyric Acid | - | - | - | I | 10 (3/31) | 0 |
| **D6** | D-Glucose- 6-PO4 | + | + | + | - | 100 | 95 (18/19) | **H6** | Acetoacetic Acid | - | - | + | I | 29 (9/31) | 16 (3/19) |
| **D7** | D-Fructose- 6-PO4 | + | + | + | + | 90 (28/31) | 42 (8/19) | **H7** | Propionic Acid | - | - | - | + | 6 (2/31) | 0 |
| **D8** | D-Aspartic Acid | - | - | - | - | 0 | 0 | **H8** | Acetic Acid | + | - | + | + | 94 (29/31) | 95 (18/19) |
| **D9** | D-Serine | + | + | I | + | 100 | 100 | **H9** | Formic Acid | + | - | - | + | 6 (2/31) | 5 (1/19) |
| **D10** | Troleandomycin | + | + | + | + | 100 | 100 | **H10** | Aztreonam | - | + | - | - | 13 (4/31) | 5 (1/19) |
| **D11** | Rifamycin SV | + | + | + | + | 100 | 100 | **H11** | Sodium Butyrate | + | + | - | + | 77 (24/31) | 26 (5/19) |
| **D12** | Minocycline | - | - | - | - | 0 | 0 | **H12** | Sodium Bromate | - | - | - | - | 32 (10/31) | 21 (4/19) |

a: LMG 3785 *A. veronii* bv. *sobria*, b: LMG 9075 *A. veronii* bv. *veronii*, c: XU 1, d: Z 1, W: West Aegean Sea, E: East Aegean Sea, (+): Positive reaction, (-): Negative reaction, I: Intermediate reaction

**Table S2.** Overview of the sequenced strains and their corresponding genome features.

|  | **NS** | **PDB** | **NS 2** | **NS 6.15.2** | **NS 13** | **NS 22** | **AG 5.28.6** | **VCK 1** | **BIOO50 A** | **XU 1** |
| --- | --- | --- | --- | --- | --- | --- | --- | --- | --- | --- |
| **Accession number** | NZ_NMUR00000000 | NZ_NMUS00000000 | NZ_NPKE00000000 | NZ_NPKC00000000 | NZ_NQMB00000000 | NZ_NQMC00000000 | NZ_NNSE00000000 | NZ_NNSF00000000 | NZ_NPKD00000000 | NZ_SSUX00000000 |
| **Status** | contig | contig | contig | contig | contig | contig | contig | contig | contig | contig |
| **Genome size (bp)** | 4.708.836 | 4.720.227 | 4.716.998 | 4.716.486 | 4.672.256 | 4.741.129 | 4.607.031 | 4.629.882 | 4.613.472 | 4.804.774 |
| **GC Content (%)** | 58,5 | 57,9 | 58,5 | 58,5 | 58,6 | 58,4 | 58,6 | 58,6 | 57,8 | 58 |
| **# Genes** | 4.551 | 4.580 | 4.575 | 4.583 | 4.525 | 4.639 | 4.380 | 4.419 | 4.417 | 4.568 |
| **# CDS** | 4.402 | 4.439 | 4.433 | 4.448 | 4.385 | 4.500 | 4.249 | 4.292 | 4.280 | 4.423 |
| **# Pseudogenes** | 177 | 200 | 224 | 246 | 187 | 235 | 144 | 165 | 203 | 91 |
| **# rRNAS** | 31 | 31 | 29 | 29 | 25 | 28 | 22 | 20 | 25 | 25 |
| **# tRNAs** | 112 | 104 | 107 | 100 | 109 | 105 | 105 | 103 | 108 | 114 |

**Table S3.** Assembly statistics of the sequenced strains.

| **Strain** | **NS** | **PDB** | **NS 2** | **NS 6.15.2** | **NS 13** | **NS 22** | **AG 5.28.6** | **VCK 1** | **BIOO50 A** | **XU 1** |
| --- | --- | --- | --- | --- | --- | --- | --- | --- | --- | --- |
| **Contigs** | 140 | 141 | 143 | 149 | 139 | 172 | 98 | 120 | 109 | 92 |
| **Total Length (bp)** | 4.708.836 | 4.720.227 | 4.716.998 | 4.716.486 | 4.672.256 | 4.741.998 | 4.607.031 | 4.629.882 | 4.613.472 | 4.804.774 |
| **Mean Length (bp)** | 33.634,54 | 33.476,79 | 32.986 | 31.654,27 | 33.613,35 | 27.564,94 | 47.010,52 | 38.582,35 | 42.325,43 | 52.789 |
| **Lower Quartile** | 3.962 | 4.234 | 3.394 | 3.056 | 3.234 | 3.117 | 8.445 | 6.298 | 7.046 | 289 |
| **Median** | 19.933 | 16.770 | 16.833 | 19.178 | 18.735 | 16.050 | 33.913 | 22.192 | 30.924 | 723 |
| **Upper Quartile** | 50.855 | 51.929 | 54.849 | 51.778 | 50.524 | 40.085 | 65.758 | 53.620 | 59.952 | 49.193 |
| **Smallest size (bp)** | 402 | 313 | 375 | 325 | 335 | 321 | 562 | 349 | 369 | 204 |
| **Biggest size (bp)** | 213.985 | 199.691 | 165.446 | 165.343 | 214.088 | 165.549 | 458.303 | 247.593 | 458.476 | 579.123 |
| **N50** | 67.042 | 72.590 | 69.902 | 66.300 | 72.418 | 61.224 | 85.872 | 68.239 | 73.700 | 206.195 |

**Table S4.** Assessment of genome assemblies using BUSCO.

| **Strain** | **Complete**  **%** | **Single copies**  **%** | **Duplicate**  **%** | **Fragmented**  **%** | **Missing %** | **# genes** |
| --- | --- | --- | --- | --- | --- | --- |
| **NS** | 94.6 | 93.9 | 0.7 | 0.0 | 5.4 | 148 |
|  | 99.3 | 98.6 | 0.7 | 0.0 | 0.7 | 148 |
| **PDB** | 94.6 | 93.9 | 0.7 | 0.0 | 5.4 | 148 |
|  | 99.3 | 98.6 | 0.7 | 0.0 | 0.7 | 148 |
| **NS 2** | 94.6 | 93.9 | 0.7 | 0.0 | 5.4 | 148 |
|  | 99.3 | 98.6 | 0.7 | 0.0 | 0.7 | 148 |
| **NS 6.15.2** | 94.6 | 93.9 | 0.7 | 0.0 | 5.4 | 148 |
|  | 99.3 | 98.6 | 0.7 | 0.0 | 0.7 | 148 |
| **NS 13** | 93.9 | 93.2 | 0.7 | 0.7 | 5.4 | 148 |
|  | 98.7 | 98.0 | 0.7 | 0.7 | 0.6 | 148 |
| **NS 22** | 94.6 | 93.9 | 0.7 | 0.0 | 5.4 | 148 |
|  | 99.3 | 98.6 | 0.7 | 0.0 | 0.7 | 148 |
| **AG 5.28.6** | 94.6 | 94.6 | 0.0 | 0.0 | 5.4 | 148 |
|  | 99.3 | 99.3 | 0.0 | 0.0 | 0.7 | 148 |
| **VCK** | 94.6 | 93.2 | 1.4 | 0.0 | 5.4 | 148 |
|  | 99.4 | 98.0 | 1.4 | 0.0 | 0.6 | 148 |
| **BIOO50 A** | 94.6 | 93.9 | 0.7 | 0.0 | 5.4 | 148 |
|  | 99.3 | 98.6 | 0.7 | 0.0 | 0.7 | 148 |
| **XU 1** | 99.3 | 98.6 | 0.7 | 0.0 | 0.7 | 148 |
|  |  |  |  |  |  |  |

Upper row: general comparison with bacteria, Lower row: Species specific

**Table S5.** Similarity of strains NS (West Aegean Sea) and VCK 1 (East Aegean Sea) with other *A. veronii* strains and *Aeromonas* species expressed as % ANI values as estimated in Ortho-ANI software.

|  | **Accession No** | **Strain** | **Isolation Source** | **Species** | **Ortho-ANI %** | |
| --- | --- | --- | --- | --- | --- | --- |
|  |  |  |  |  | NS | VCK |
| 1 | CP012504 | TH0426 | *Pelteobagrus fulvidraco* | *A. veronii* | 96,3 | 96,4 |
| 2 | CP015448 | CB51 | grass carp | *A. veronii* | 96,2 | 96,2 |
| 3 | CP024930 | X11 | *Megalobrama amblycephala* | *A. veronii* | 96,3 | 96,4 |
| 4 | CP024933 | X12 | *Megalobrama amblycephala* | *A. veronii* | 96,3 | 96,3 |
| 5 | CP028133 | 17ISAe | *Symphysodon discus* | *A. veronii* | 96,2 | 96,3 |
| 6 | NZ_PZKL00000000 | XH.VA.1 | *Ictalurus punctatus* | *A. veronii* | 96,2 | 96,3 |
| 7 | NZ_RAWX00000000 | MS 17-88 | catfish | *A. veronii* | 96,3 | 96,3 |
| 8 | NZ_CP033604 | MS-18-37 | catfish | *A. veronii* | 96,4 | 96,5 |
| 9 | NZ_ATFB00000000 | Hm21 | *Hirudo verbana*-digestive tract | *A. veronii* | 96,5 | 96,5 |
| 10 | RZII00000000 | CQ-AV1 | *Andrias davidianus* (Chinese giant salamander) | *A. veronii* | 96,2 | 96,3 |
| 11 | NC_015424 | B565 | aquaculture pond sediment | *A. veronii* | 96,4 | 96,5 |
| 12 | NZ_NJGB00000000 | A29 | surface water | *A. veronii* | 96,3 | 96,4 |
| 13 | NZ_CDBU00000000 | CECT 4486 | surface water | *A. veronii* | 96,2 | 96,2 |
| 14 | NZ_CDBQ00000000 | LMG 13067 | emvironmental | *A. veronii* | 96,5 | 96,6 |
| 15 | CP014774 | AVNIH1 | Human-perirectal culture | *A. veronii* | 96,3 | 96,3 |
| 16 | CP032839 | FC951 | Human-feces | *A. veronii* | 96,2 | 96,3 |
| 17 | NZ_PPTE00000000 | 126-14 | stool sample - Homo sapiens | *A. veronii* | 96,4 | 96,4 |
| 18 | NZ_JH823256 | AMC34 | Human Microbiome Project (HMP) | *A. veronii* | 93,8 | 93,9 |
| 19 | NZ_AGWT00000000 | AER39 | Human Microbiome Project (HMP) | *A. veronii* | 96,4 | 96,4 |
| 20 | NZ_CDDK00000000 | CECT 4257 | sputum of drowning victim-human | *A. veronii* | 96,4 | 96,5 |
| 21 | NZ_LKJN00000000 | TTU2014-108AME | dairy cattle | *A. veronii* | 96,3 | 96,4 |
| 22 | NZ_NXBU00000000 | Z2-7 | pork | *A. veronii* | 96,2 | 96,3 |
| 23 | NZ_SSUX00000000 | XU 1 | *Xiphophorus helleri* | *A. veronii* | 96,4 | 96,5 |
| 24 | NC_008570 | ATCC 7966 | tin of milk with a fishy odor | *A. hydrophila* subsp*. hydrophila* | 84,5 | 85,8 |
| 25 | CP000644 | A449 | *Salmo trutta* (brown trout) | *A. salmonicida* subsp. *salmonicida* | 84,5 | 86,0 |

**Table S6.** The outer membrane proteins detected in the nine *A. veronii* strains from seabass. Locus tag refers to the genome of strain VCK 1. The score for outer membrane localization from PSORTb, the type of signal peptides (SPI and SPII) of the general secretion pathway (Sec) detected from Signal IP, the number of transmembrane (TM) α-helices and β-strands detected by TOPCONS and PRED-TMBB2 respectively are presented. Homology in 3D structure with proteins deposited in Protein Data Bank (PDB) as detected through the analysis in TOPCONS is also presented. Protein sequence distances are presented as % similarity among seabass strains (W/E) and between seabass strains and strain B565 (*A. veronii*) and XU 1.

**Table S7.** The genes of virulence factors as detected manually and through PATRIC in the nine seabass strains. The genes of secretion systems, secreted proteins and toxins and clusters containing flagellar genes are presented through the locus tags of the corresponding CDSs.
