# Supplementary figures and images for: Comparative Study on A Novel Pathogen of European Seabass. Diversity of Aeromonas veronii in the Aegean Sea
Source: Microorganisms. 2019 Oct 29;7(11):504. doi: 10.3390/microorganisms7110504 (PMC6921072; doi:10.3390/microorganisms7110504)

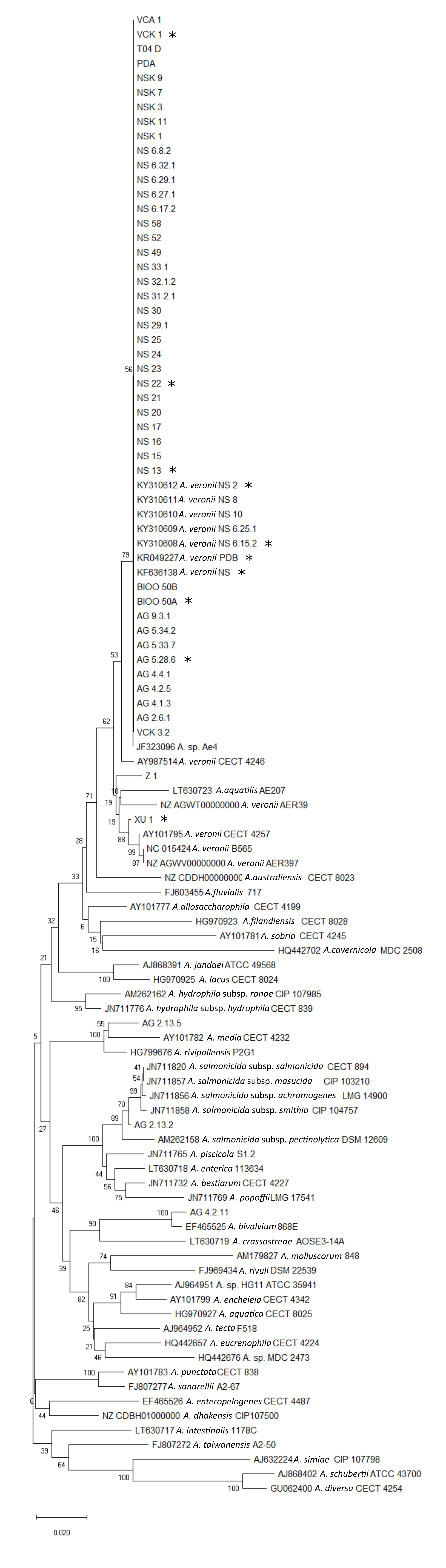

Supplement: Supplementary file 1 [file microorganisms-07-00504-s001.zip › Figure S1.png]
